# Effect of Yttrium Addition on Structure and Magnetic Properties of Co_60_Fe_20_Y_20_ Thin Films

**DOI:** 10.3390/ma14206001

**Published:** 2021-10-12

**Authors:** Wen-Jen Liu, Yung-Huang Chang, Yuan-Tsung Chen, Ding-Yang Tsai, Pei-Xin Lu, Shih-Hung Lin, Te-Ho Wu, Po-Wei Chi

**Affiliations:** 1 Department of Materials Science and Engineering, I-Shou University, Kaohsiung 840, Taiwan; jurgen@isu.edu.tw; 2 Bachelor Program in Interdisciplinary Studies, National Yunlin University of Science and Technology, 123 University Road, Section 3, Yunlin 64002, Taiwan; changyhu@yuntech.edu.tw; 3 Graduate School of Materials Science, National Yunlin University of Science and Technology, 123 University Road, Section 3, Yunlin 64002, Taiwan; M10847010@yuntech.edu.tw (D.-Y.T.); M11047015@yuntech.edu.tw (P.-X.L.); wuth@yuntech.edu.tw (T.-H.W.); 4 Department of Electronic Engineering, National Yunlin University of Science and Technology, 123 University Road, Section 3, Yunlin 64002, Taiwan; isshokenmei@yuntech.edu.tw; 5 Institute of Physics, Academia Sinica, Nankang, Taipei 11529, Taiwan; jacky01234567891@hotmail.com

**Keywords:** annealed Co_60_Fe_20_Y_20_ thin films, saturation magnetization (Ms), low-frequency alternating current magnetic susceptibility (χ_ac_), optimal resonance frequency (f_res_), surface energy, adhesion

## Abstract

In this paper, a Co_60_Fe_20_Y_20_ film was sputtered onto Si (100) substrates with thicknesses ranging from 10 to 50 nm under four conditions to investigate the structure, magnetic properties, and surface energy. Under four conditions, the crystal structure of the CoFeY films was found to be amorphous by an X-ray diffraction analyzer (XRD), suggesting that yttrium (Y) added into CoFe films and can be refined in grain size and insufficient annealing temperatures do not induce enough thermal driving force to support grain growth. The saturation magnetization (M_S_) and low-frequency alternate-current magnetic susceptibility (χ_ac_) increased with the increase of the thicknesses and annealing temperatures, indicating the thickness effect and Y can be refined grain size and improved ferromagnetic spin exchange coupling. The highest Ms and **χ_ac_** values of the Co_60_Fe_20_Y_20_ films were 883 emu/cm^3^ and 0.26 when the annealed temperature was 300 °C and the thickness was 50 nm. The optimal resonance frequency (f_res_) was 50 Hz with the maximum χ_ac_ value, indicating it could be used at a low frequency range. Moreover, the surface energy increased with the increase of the thickness and annealing temperature. The maximum surface energy of the annealed 300 °C film was 30.02 mJ/mm^2^ at 50 nm. Based on the magnetic and surface energy results, the optimal thickness was 50 nm annealed at 300 °C, which has the highest Ms, χ_ac_, and a strong adhesion, which can be as a free or pinned layer that could be combined with the magnetic tunneling layer and applied in magnetic fields.

## 1. Introduction

A CoFe binary alloy is an important alloy with excellent soft magnetic properties. It has a unique combination of high saturation magnetization (M_S_), a high Curie temperature (T_C_), low magnetic crystal anisotropy, and good strength. It is suitable for applications requiring a high-throughput density. Due to these excellent characteristics, researchers often apply CoFe alloys to magnetic equipment, and they can be used in micro electromechanical systems (MEMS), sensors, actuators, and magnetic recording heads [1,2,3]. However, CoFe alloys have the disadvantage of serious magnetic degradation and anisotropy with the increase of temperature after annealing treatment. 

Therefore, it is difficult for these magnetic devices to be effectively used at high temperatures. In order to enhance the thermal stability of CoFe alloys, a third element may be added to overcome this problem [4]. Therefore, the main research goal of this study was to find ways to effectively improve the thermal stability of CoFe alloys by adding other elements. Magnetic films play an important role in increasing the storage density of magnetic data. In this region, the manufacturing of write heads requires high M_S_, low coercivity (H_C_), and high anisotropy [5]. 

However, when rare earth elements and transition metals, such as yttrium (Y), iron (Fe), cobalt (Co), and nickel (Ni), are combined to form compounds, their Curie temperatures will be higher than room temperature (RT). Therefore, their characteristics can be applied to improve the high temperature resistance of magnetic films and improve their mechanical strength and other physical properties. CoFeY is a new material in the field of magnetic materials. 

The chemical properties of yttrium are similar to those of the lanthanides (Ln), and it is often classified as a rare earth metal. In the past, Y was used as a reactant for phosphors to produce various synthetic garnets. According to the literature, the addition of Y to the parent alloy and heat treatment can significantly improve the mechanical properties [6]. The addition of Y improves the thermal stability and corrosion resistance [7]. The addition of Y into an alloy can reduce the processing difficulty, improve the recrystallization of the material at high temperatures, and greatly increase the resistance to high temperature oxidation [8,9]. 

In addition, the substitution of Y can not only enhance the exchange coupling but also improve the stability of the magnetic properties and temperature [10,11]. Regarding the application of Y in soft magnetic materials, since 2009, some scholars have successfully developed large-size ternary Fe–X–B (X = S_C_, Y, Dy, Ho, and Er) amorphous alloys with a small amount of rare earth elements [12,13]. Due to the advantage of having a low cost in industrial application, Fe–Y–B also has relatively high potential. Thus far, studies have shown that adding or increasing the content of Y in permanent magnet materials can reduce their coercivity and improve their thermal stability. 

However, there are few studies on adding Y to soft magnetic materials. For Fe–Y–B alloys, it is necessary to increase the soft magnetic properties, such as low-temperature annealing treatment [14,15]. It is worthwhile to study the addition of pure rare earth elements to transition alloys to investigate the resulting characteristics, including the structure, magnetic properties, surface energy, and electric resistivity. As there are few studies focused on Co_60_Fe_20_Y_20_ films, we focused on the use of Co_60_Fe_20_Y_20_ alloys on a Si (100) substrate. 

We discuss whether the addition of Y could improve the characteristics and magnetic properties of a magnetic film, such as its thermal stability and comparison at room temperature. In this experiment, the structure, magnetic properties, and adhesion were discussed under four conditions: at as-deposited temperature and after annealing at 100, 200, and 300 °C. The specific properties Co_60_Fe_20_Y_20_ films can be compared with other specific Co_75_Fe_25_ films using their magnetic characteristics, as mentioned in Table 1 [16,17]. The results revealed that the addition of Y can enhance the exchange coupling and grain refinement, thus, inducing higher saturation magnetization and lower coercivity.

## 2. Materials and Methods

CoFeY with thickness of 10–50 nm was sputtered onto a Si(100) substrate at RT by magnetron direct current (DC) sputtering method of 50 W power and under the subsequent four conditions: (a) the deposited films were kept at RT, (b) annealed at a treatment temperature (T_A_) at 100 °C for 1 h, (c) annealed at 200 °C for 1 h, and (d) annealed at 300 °C for 1 h. The chamber base pressure was 3 × 10^−7^ Torr, and therefore the Ar working pressure was 5 × 10^−3^ Torr. The pressure in ex-situ annealed condition was 3 × 10^−3^ Torr with a selected Ar gas. The alloy target of CoFeY composition was Co 54.7 wt%, Fe 17.5 wt%, and Y 27.7 wt%. 

To achieve accurate thicknesses, the calibrated thickness of the corresponding sputtered time was investigated through high-resolution cross-sectional transmission electron microscopy (HR X-TEM) (JEOL, Tokyo, Japan). The structure was detected by grazing incidence X-ray Diffraction (XRD) (Malvern Panalytical Ltd, Cambridge, United Kingdom) patterns obtained with CuKα1 (PAN analytical X’pert PRO MRD, Malvern Panalytical Ltd, Cambridge, United Kingdom) and a low angle diffraction incidence of about a two-degree angle. In-plane low-frequency alternate-current magnetic susceptibility (χ_ac_) and hysteresis loops were studied by χ_ac_ analyzer (XacQuan, MagQu Co. Ltd. New Tapei City, and Taiwan) and alternating gradient magnetometer (AGM) (PMC, Ohio, USA).

Furthermore, in the χ_ac_ measurement, we used the χ_ac_ analyzer to calibrate the standard sample under the action of an external magnetic field and inserted the sample into the χ_ac_ analyzer. The driving frequency was between 10 and 25,000 Hz. χ_ac_ was measured by magnetization. All test samples had an equivalent shape and size to eliminate demagnetization. The χ_ac_ valve was an arbitrary unit (a.u.) because the AC result corresponds to the reference standard sample and may be a comparison value. The connection between magnetic susceptibility and frequency was measured by a χ_ac_ analyzer. 

The best resonance frequency (f_res_) was measured by a χ_ac_ analyzer and represents the frequency of the maximum χ_ac_. Before measurement, the contact angle should be properly air cleaned on the surface. The contact angles of CoFeY film were measured with deionized (DI) water and glycerol. The contact angles were measured when the samples take out from the chamber. The surface energy was obtained from the contact angle and some calculations [18,19,20]. The resistivity (ρ) was measured using a conventional four-point technique.

## 3. Results

### 3.1. Structure

**Figure 1:**
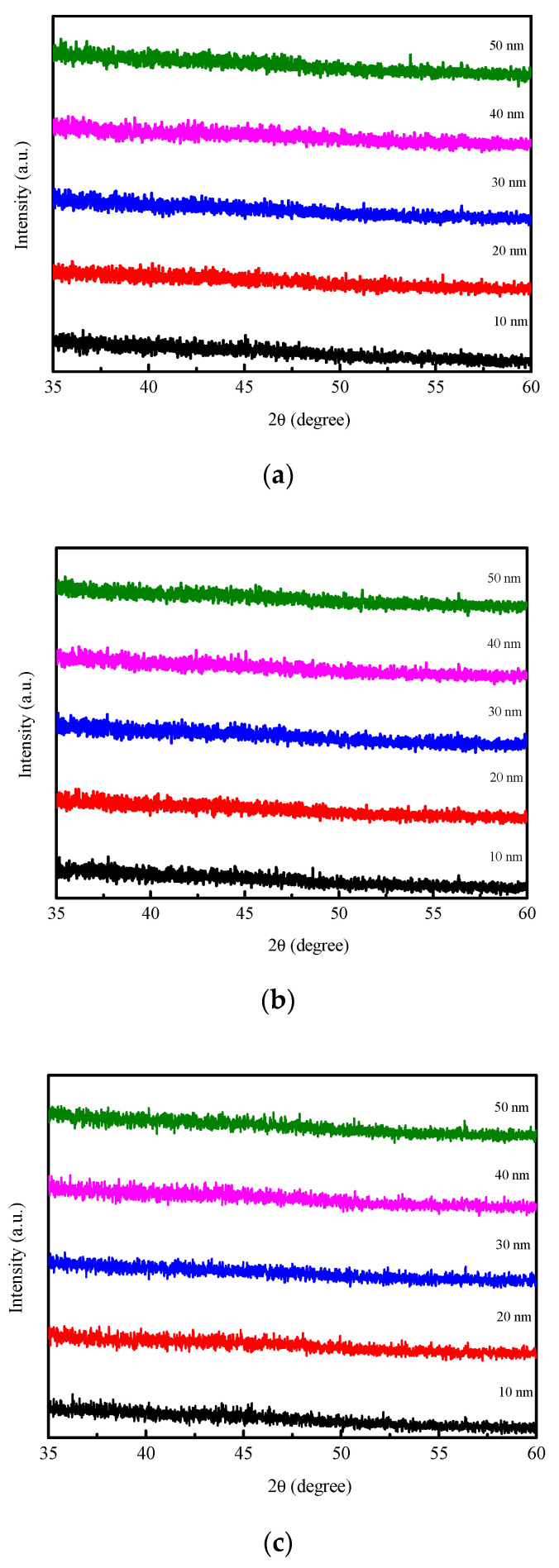

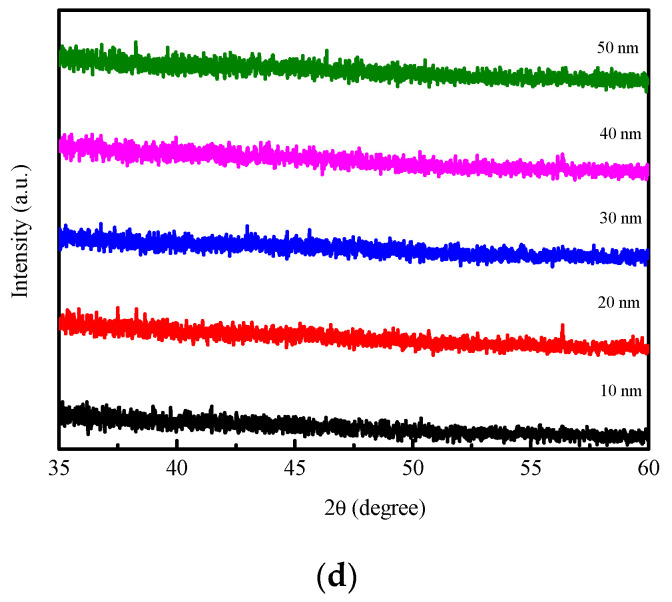
X-ray diffraction patterns of CoFeY thin films. (**a**) RT, (**b**) post-annealing at 100 °C, (**c**) post-annealing at 200 °C, and (**d**) post-annealing at 300 °C.

Figure 1a–d display the XRD results of the CoFeY films at four conditions. To avoid Si(100) substrate signal is too strong to cause other peak signals to be invisible in the particular range of diffraction angles, the XRD patterns of the different samples detected in the range from 35 to 60 degrees. Moreover, XRD patterns also subtracted the background signal from the Si substrate. From the results of Figure 1a, there was no apparent diffracted peak, indicating an amorphous structure of the as-deposited film. Moreover, after the annealing treatments, an amorphous state was found, because Y was added to the CoFe alloy and could refine the grain size as shown in Figure 1b–d [21,22,23]. Another possible reason is the lack of a sufficient thermal driving force to support grain growth [24,25,26].

### 3.2. Magnetic Analysis

**Figure 2:**
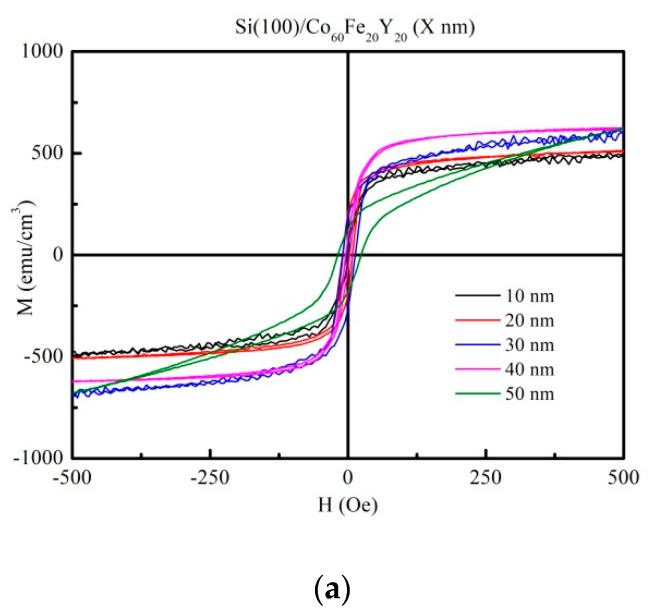

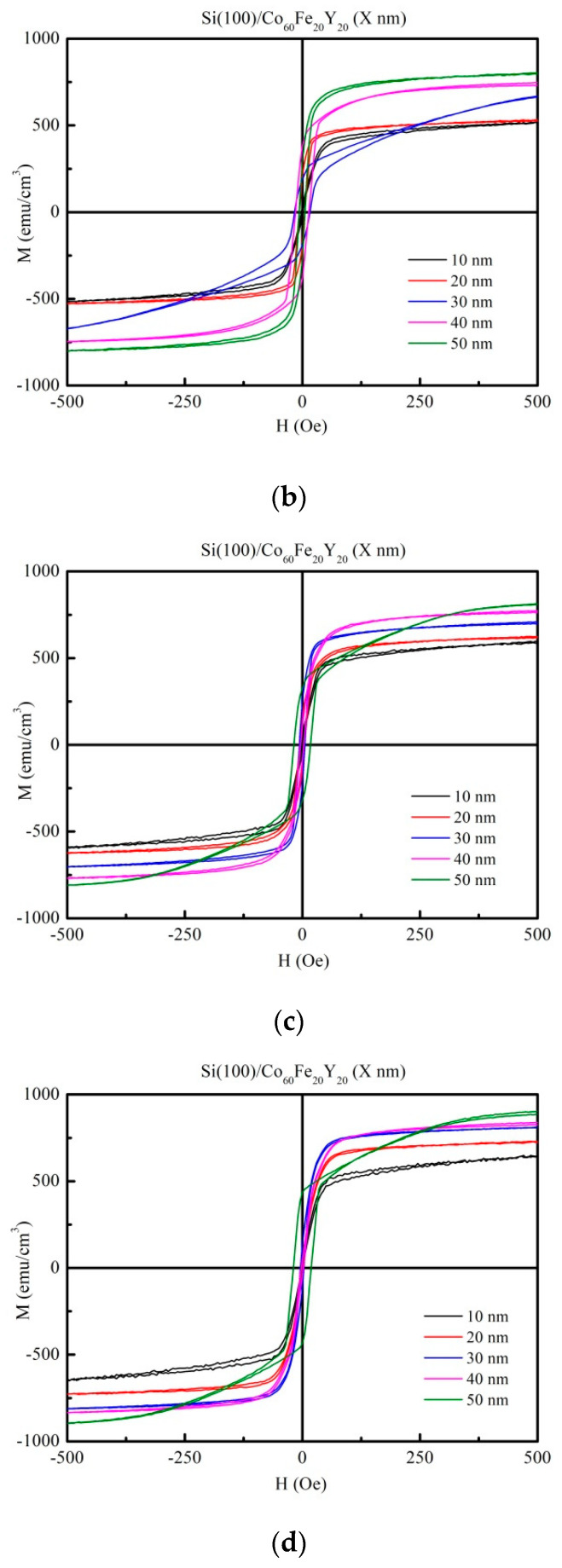
In-plane magnetic hysteresis loop of CoFeY thin films. (**a**) RT, (**b**) post-annealing at 100 °C, (**c**) post-annealing at 200 °C, and (**d**) post-annealing at 300 °C.

Figure 2a–d reveal the in-plane hysteresis loops of the CoFeY films under four conditions, with thicknesses ranging from 10 to 50 nm as measured using AGM. The external magnetic field (H_ext_) of 500 Oe in the plane was enough to observe the saturation magnetic spin state. The figures reveal low coercivity, suggesting the CoFeY film had soft magnetic properties.

**Figure 3:**
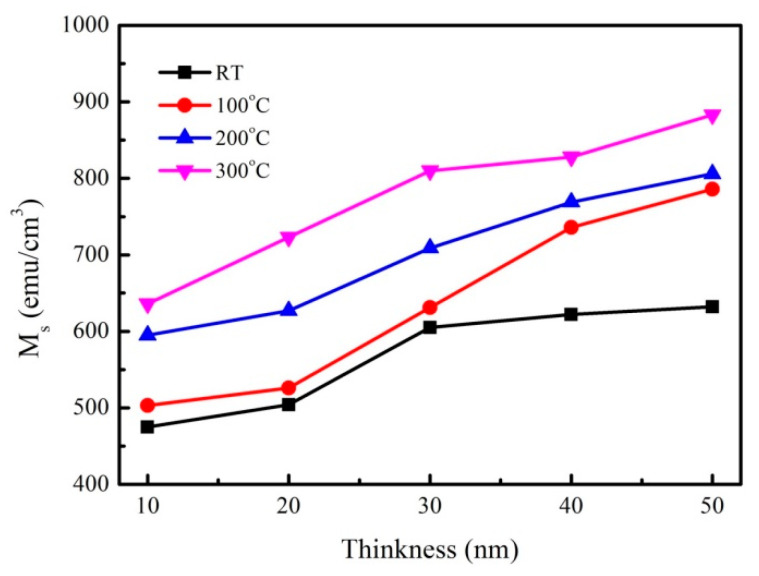
Saturation magnetization (M_S_) of CoFeY thin films.

The corresponding saturation magnetization (Ms) of the CoFeY various thicknesses under four conditions are shown in Figure 3. From the result of Figure 3, the Ms increased with the increase of the thickness and annealing temperature owing to the thickness effect and the addition of Y can enhance the exchange coupling and grain refinement. The ferromagnetic spin exchange coupling is improved [27,28,29]. The highest saturation magnetization occurred at the annealing temperature of 300 °C, indicating that the best heat-resistant temperature of the films in this study was 300 °C. 

The maximum Ms of the CoFeY films at 50 nm under four conditions was 632, 786, 806, and 883 emu/cm^3^, respectively. The results showed that the addition of Y and the annealing temperature could increase the magnetization and improve the properties of the alloy. The saturation magnetization of the samples increased roughly by 50% in its value, when the samples were annealed to 300 °C. The structure of the films has not changed. It can be reasonably concluded that the increase in magnetization was caused by the annealing treatment [30].

**Figure 4:**
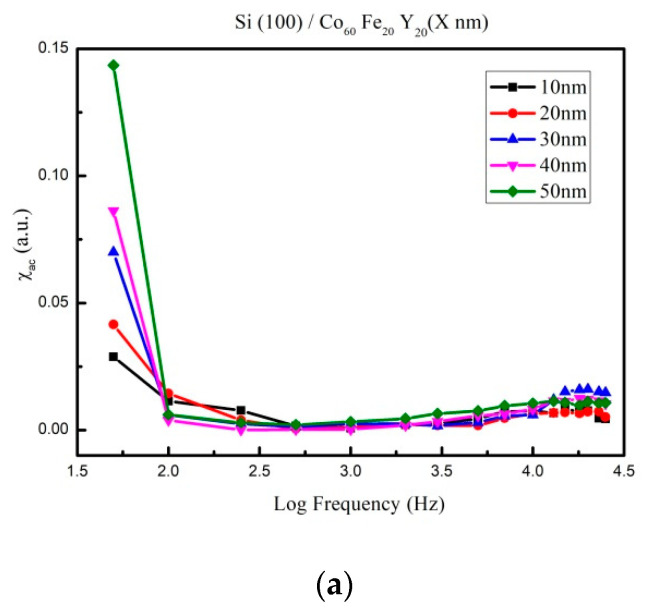

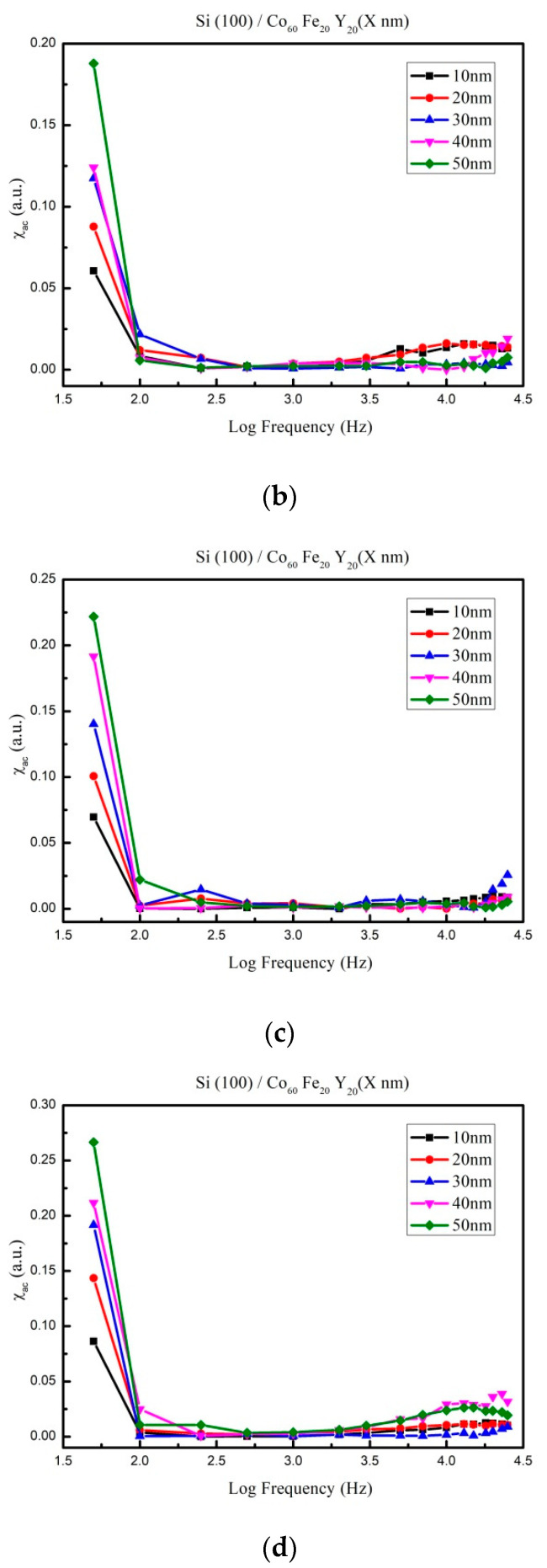
The low-frequency alternate-current magnetic susceptibility (χ_ac_) as a function of the frequency from 50 to 25,000 Hz. (**a**) RT, (**b**) post-annealing at 100 °C, (**c**) post-annealing at 200 °C, and (**d**) post-annealing at 300 °C.

**Figure 5:**
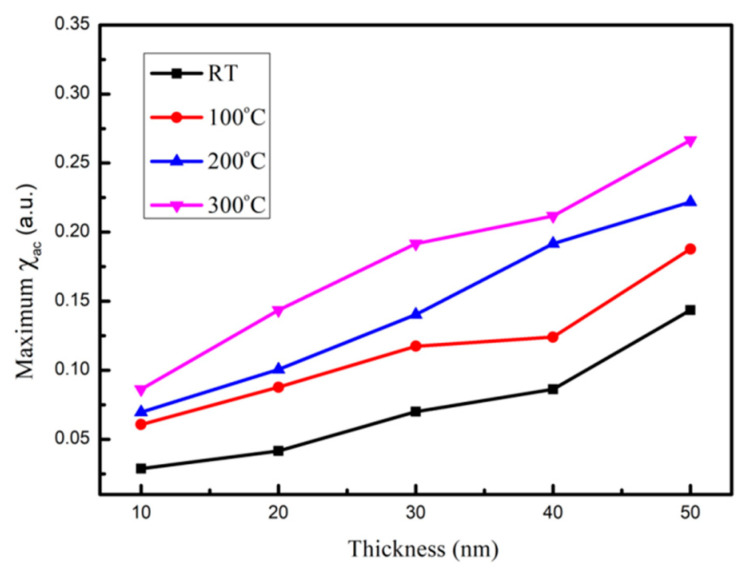
The maximum alternate-current magnetic susceptibility for the CoFeY thin films.

Figure 4a–d depict the low-frequency alternating-current magnetic susceptibility (χ_ac_) as a function of 50 to 25000 Hz at 10, 20, 30, 40, and 50 nm under four conditions. The frequency in the X-axis represented on a logarithmic scale, which is shown in Figure 4. Apparently, maximum χ_ac_ of all CoFeY films is found at 50 Hz. The χ_ac_ values decreased sharply with the increasing frequency under four conditions. The corresponding maximum χ_ac_ of various CoFeY thicknesses under four conditions are shown in Figure 5. According to the results, the maximum χ_ac_ also increased with the increase of the thickness and annealing temperature owing to the thickness effect, and the addition of Y can enhance the exchange coupling and grain refinement, indicating that the ferromagnetic spin exchange coupling is improved [27,28,29]. 

The χ_ac_ peak indicated the spin exchange-coupling interaction and dipole moment of the domain under frequency [31]. The maximum χ_ac_ of the CoFeY films at 50 nm under four conditions was 0.14, 0.18, 0.22, and 0.26, respectively. The maximum χ_ac_ demonstrated that the spin sensitivity was highest at the optimal resonant frequency (f_res_). Furthermore, the ƒ_res_ value was 50 Hz for all films, making CoFeY films ideal for low-frequency magnetic applications in soft magnetism devices.

### 3.3. Analysis of Surface Energy and Adhesion

The contact angles of the CoFeY films were measured using DI water and glycerol under the four conditions, as shown in Table 2. The contact angles of all Co_60_Fe_20_Y_20_ thin films were less than 90°, indicating that the CoFeY films exhibited good hydrophilicity and wettability. The result revealed that, as the annealed temperature increased, the contact angle showed a decreasing trend. The surface energy of a thin film is an important parameter because it relates to the adhesion of thin films. When CoFeY thin films are used as a seed, buffer, free, or pinned layer, the strong adhesion of the thin films is essential. The data of the contact angles are used to calculate the surface energy [18,19,20].

**Figure 6:**
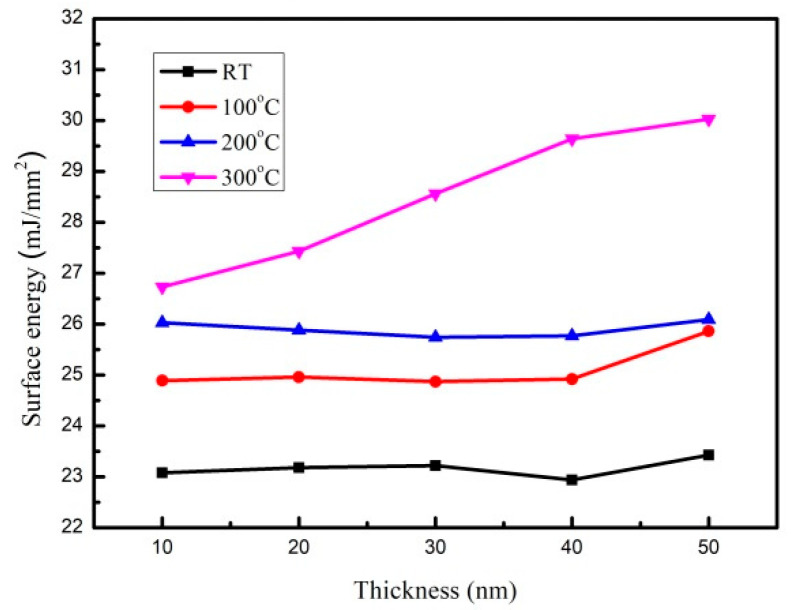
Surface energy of CoFeY films.

Figure 6 shows the trend of the CoFeY surface energy, and the value is shown in Table 2. The surface energy of the CoFeY film increased with the increase of the thickness. Apparently, the surface energy of the CoFeY film increased with the annealing temperature. The highest surface energy of annealed 100 °C CoFeY thin films was 25.86 mJ/mm^2^ at 50 nm. When the post-annealing temperature was 200 °C, the highest surface energy at 50 nm was 26.09 mJ/mm^2^. 

When the post-annealing temperature achieved 300 °C, it was 30.02 mJ/mm^2^ at 50 nm. When the surface energy was high, the liquid absorption capacity of the surface was correspondingly high. A high surface energy corresponds to strong adhesion [32]. Surface energy is an important factor affecting the adhesion of a film. As CoFeY is compatible with the spin-value magnetic tunnel junction (MTJ) and can be applied to magnetoresistance random access memory (MRAM) applications, it can also be used as a free layer and in combination with other layers.

### 3.4. Electric Property

**Figure 7:**
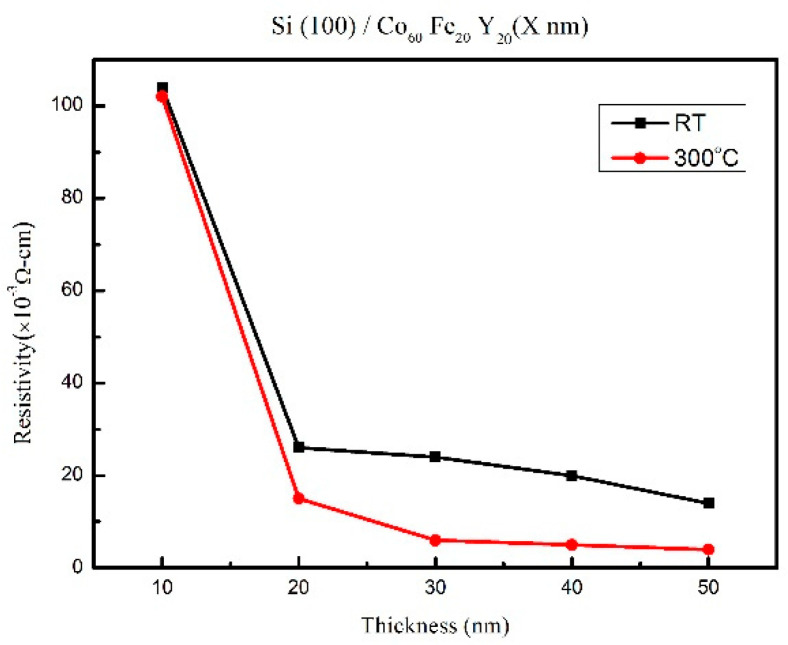
Resistivity of CoFeY films.

The electric resistivity (ρ) is shown in Figure 7. From the results, the resistivity decreased with the increase of thickness and annealed temperatures. The electrical resistivity of the as-deposited and thinner CoFeY films was larger than that of the annealed and thicker CoFeY films because the as-deposited and thinner CoFeY films decreased the electron mobility through the film and enhanced the electron scattering at the grain boundaries or where impurities are present [33,34].

## 4. Conclusions

The XRD results showed that the CoFeY had no obvious characteristic peak, and thus CoFeY has an amorphous structure owing to the grain size refinement of Y addition and insufficient thermal driving force for grain growth. Ms and χ_ac_ both increased with the increase of the thickness and the annealing temperature owing to the thickness effect, and the addition of Y can enhance the exchange coupling and grain refinement. The ferromagnetic spin exchange coupling was improved. 

From the magnetic measurement results, we that, when the film thickness was 50 nm and the annealing temperature was 300 °C, the saturated magnetization M_S_ and χ_ac_ values of CoFeY films were the highest, at 883 emu/cm^3^ and 0.26. The best resonance frequency of the CoFeY film was 50 Hz, indicating that it could be used in low-frequency applications. Moreover, the contact angle of the surface was less than 90°, indicating that it was a hydrophilic film. The surface energy of the CoFeY film increased with the increase of the thickness and the annealing temperature. 

At 50 nm of annealing at 300 °C, the film had the highest surface energy of 30.02 mJ/mm^2^ and had the strongest adhesion. The higher surface energy resulted in the stronger adhesion of the film, and it was easier to combine with magnetic tunneling layer to form an MTJ structure. Based on the magnetic and surface energy results, the optimal thickness was 50 nm at an annealing temperature of 300 °C. This had the highest Ms, χ_ac_, and strong adhesion, making it useful as a free or pinned layer that could be combined with the magnetic tunneling layer and applied in magnetic fields.

## Figures and Tables

**Table 1:** Significant properties for Co_60_Fe_20_Y_20_ and Co_75_Fe_25_ materials.

Material	Saturation Magnetization Ms (emu/cm^3^)	Coercivity Hc (Oe)	Magnetic Susceptibility χ (arb. units)
CoFe alloy nanoparticles [16]	197–213	177–296	
Cu (110)/Co_75_Fe_25_ (0.5–1 ML) [17]			0–1.2
Si (100)/Co_60_Fe_20_Y_20_ [*] 10–50 nm at as-deposited and annealed conditions	475–883	1–21	0.03–0.26

[*]: Current research.

**Table 2:** Comparing the contact angles and surface energy for Co_60_Fe_20_Y_20_ thin films from different fabrication processes.

Process	Thickness	Contact Angle with DI Water (θ)	Contact Angle with Glycerol (θ)	Surface Energy (mJ/mm^2^)
RT	10 nm	82.7°	80.0°	23.08
	20 nm	81.8°	80.2°	23.18
	30 nm	82.9°	78.7°	23.22
	40 nm	83.4°	80.5°	22.94
	50 nm	83.6°	77.9°	23.43
Post-annealing 100 °C	10 nm	81.5°	75.7°	24.89
	20 nm	80.1°	76.9°	24.96
	30 nm	80.2°	77.6°	24.87
	40 nm	83.2°	75.7°	24.92
	50 nm	79.2°	78.2°	25.86
Post-annealing 200 °C	10 nm	82.5°	74.3°	26.03
	20 nm	78.9°	75.8°	25.88
	30 nm	79.1°	77.5°	25.74
	40 nm	83.7°	75.0°	25.77
	50 nm	78.7°	77.4°	26.09
Post-annealing 300 °C	10 nm	77.9°	74.1°	26.73
	20 nm	79.7°	79.5°	27.43
	30 nm	77.7°	72.2°	28.56
	40 nm	74.0°	72.0°	29.64
	50 nm	73.5°	69.9°	30.02

## Data Availability

The data presented in this study are available on reasonable request from the corresponding author.

## References

[1] Sunder R.S., Deevi S.C. (2005). Soft magnetic FeCo alloys: Alloy development, processing, and properties. Int. Mater. Rev..

[2] Hunter D., Osborn W., Wang K., Kazantseva N., Hattrick-Simpers J., Suchoski R., Takahashi R., Young M.L., Mehta A., Bendersky L.A. (2011). Giant magnetostriction in annealed Co_1–x_Fe_x_ thin-films. Nat. Commun..

[3] Zhang H., Tang X., Wei R., Zhu S., Yang J., Song W., Dai J., Zhu X., Sun Y. (2017). Microstructure refinement and magnetization improvement in CoFe thin films by high magnetic field annealing. J. Alloys Compd..

[4] Kockar H., Ozergin E., Karaagac O., Alper M. (2014). Characterisations of CoFeCu films: Influence of Fe concentration. J. Alloys Compd..

[5] Mehrizi S., Sohi M.H., Ebrahimi S.A.S. (2011). Study of microstructure and magnetic properties of electrodeposited nanocrystalline CoFeNiCu thinfilms. Surf. Coat. Technol..

[6] Cui C., Wu L., Wu R., Zhang J., Zhang M. (2011). Influence of yttrium on microstructure and mechanical properties of as-cast Mg–5Li–3Al–2Zn alloy. J. Alloys Compd..

[7] Won S., Seo B., Park H.K., Kim H.K., Kang H.S., Park K. (2021). Impact of Yttrium on corrosion properties of titanium as a grain refiner. Mater. Today Commun..

[8] Baulin O., Bugnet M., Fabregue D., Lenain A., Gravier S., Cazottes S., Kapelski G., Ovanessian B.T., Balvay S., Hartmann D.J. (2018). Improvement of mechanical, thermal, and corrosion properties of Ni- and Al-free Cu–Zr–Ti metallic glass with yttrium addition. Materialia.

[9] Guo Y., Jia L., Zhang H., Zhang F., Zhang H. (2018). Enhancing the oxidation resistance of Nb-Si based alloys by yttrium addition. Intermetallics.

[10] Liu Z.W., Qian D.Y., Zhao L.Z., Zheng Z.G., Gao X.X., Ramanujan V.R. (2014). Enhancing the coercivity, thermal stability and exchange coupling of nano-composite (Nd, Dy, Y)–Fe–B alloys with reduced Dy content by Zr addition. J. Alloys Compd..

[11] Liu S.Y., Cao Q.P., Wang X.D., Zhang D.X., Su Q.M., Du G.H., Jiang J.Z. (2016). Effects of thickness on structure and magnetic property of Fe-Y-B thin films. Thin Solid Films.

[12] Makino A., Chang C., Kubota T., Inoue A. (2009). Soft magnetic Fe-Si-B-P-C bulk metallic glasses without any glass-forming metal elements. J. Alloys Compd..

[13] Gu Z., Ma D., Xu C., Liu T., Cheng L., Du Y., Zhang W. (2018). Crystal structure and phase relations of the R2Fe14B–Y2Fe14B (R=Nd and Pr) systems. J. Supercond. Nov. Magn..

[14] Liu S.Y., Cao Q.P., Mori S., Ishigami K., Igarashi K., Wang C., Qian X., Wang X.D., Zhang D.X., Riedmullaer B. (2014). Synthesis and magnetic properties of amorphous Fe-Y-B thin films. J. Alloys Compd..

[15] Huang X.M., Wang X.D., Jiang J.Z. (2009). Origin of high glass forming ability of Y-containing FeB-based alloys. J. Alloys Compd..

[16] Yang S., Wang L., Yue S., Lu Y., He J., Zhao D. (2014). Influence of reduction temperature on composition, particle size, and magnetic properties of CoFe alloy nanomaterials derived from layered double hydroxide precursors. Dalton Trans..

[17] Küpper D., Easton S., Bland J.A.C. (2007). Paramagnetic-ferromagnetic phase transition and magnetic properties of ultrathin CoFe/Cu(110) films. J. Appl. Phys..

[18] Ma K., Chung T.S., Good J.R. (1998). Surface energy of thermotropic liquid crystalline polyesters and polyesteramide. J. Polym. Sci..

[19] Owens D.K., Wendt R.C. (1969). Estimation of the surface free energy of polymers. J. Appl. Polym. Sci..

[20] Kaelble D.H., Uy K.C. (1970). A Reinterpretation of Organic Liquid-Polytetrafluoroethylene Surface Interactions. J. Adhes..

[21] Wu Y., Hwang S.K. (2002). Microstructural refinement and improvement of mechanical properties and oxidation resistance in EPM TiAl-based intermetallics with yttrium addition. Acta Mater..

[22] Korgiopoulos K., Pekguleryuz M. (2020). The significant effect of trace yttrium level on the mechanical properties of cast Mg–6Al alloy through a refinement mechanism. Mater. Sci. Eng. A.

[23] Wang H.Y., Zhu J.N., Li J.H., Li C., Zha M., Wang C., Yang Z.Z., Jiang Q.C. (2017). Refinement and modification of primary Mg_2_Si in an Al–20Mg_2_Si alloy by a combined addition of yttrium and antimony. Cryst. Eng. Comm..

[24] Eckert J., Holzer J.C., Johnson W.L. (1993). Thermal stability and grain growth behavior of mechanically alloyed nanocrystalline Fe-Cu alloys. J. Appl. Phys..

[25] Kube S.A., Xing W., Kalidindi A., Sohn S., Datye A., Amram D., Schuh C.A., Schroer J. (2020). Combinatorial study of thermal stability in ternary nanocrystalline alloys. Acta Mater..

[26] Peng H.R., Huang L.K., Liu F. (2018). A thermo-kinetic correlation for grain growth in nanocrystalline alloys. Mater. Lett..

[27] Chen Z., Luo J., Sui Y., Guo Z. (2010). Effect of yttrium substitution on magnetic properties and microstructure of Nd-Y-Fe-B nanocomposite magnets. J. Rare Earth..

[28] Liu Z., Qian D., Zeng D. (2012). Reducing Dy Content by Y Substitution in Nanocomposite NdFeB Alloys With Enhanced Magnetic Properties and Thermal Stability. IEEE Trans. Magn..

[29] Zhang M., Zhang Z.D., Sun X.K., Liu W., Geng D.Y., Jin X.M., You C.Y., Zhao X.G. (2002). Beneficial effect of nonmagnetic Y on magnetic properties due to the enhancement of exchange coupling in nanocomposite (Nd,Y)_2_Fe_14_B/α–Fe magnets. J. Appl. Phys..

[30] Ueda K., Tan A.J., Beach D.G.S. (2018). Effect of annealing on magnetic properties in ferrimagnetic GdCo alloy films with bulk perpendicular magnetic anisotropy. AIP Adv..

[31] Yang S.Y., Chien J.J., Wang W.C., Yu C.Y., Hing N.S., Hong H.E., Hong C.Y., Yang H.C., Chang C.F., Lin H.Y. (2011). Magnetic nanoparticles for high-sensitivity detection on nucleic acids via superconducting-quantum-interference-device-based immunomagnetic reduction assay. J. Magn. Magn. Mater..

[32] Porter D.A., Easterling K.E. (1992). Phase Transformations in Metals and Alloy.

[33] Jassim S.A.J., Zumaila A.A.R.A., Waly G.A.A.A. (2013). Influence of substrate temperature on the structural, optical and electrical properties of CdS thin films deposited by thermal evaporation. Results Phys..

[34] Redjdal N., Salah H., Hauet T., Menari H., Chérif S.M., Gabouze N., Azzaz M. (2014). Microstructural, electrical and magnetic properties of Fe_35_Co_65_ thin films grown by thermal evaporation from mechanically alloyed powders. Thin Solid Films.

